# The Relationship between Bipolar Disorder and Cannabis Use in Daily Life: An Experience Sampling Study

**DOI:** 10.1371/journal.pone.0118916

**Published:** 2015-03-04

**Authors:** Elizabeth Tyler, Steven Jones, Nancy Black, Lesley-Anne Carter, Christine Barrowclough

**Affiliations:** 1 The Spectrum Centre for Mental Health Research, Division of Health Research Lancaster University, Lancaster, Lancashire, United Kingdom; 2 School of Psychological Sciences, University of Manchester, Manchester, United Kingdom; 3 Health Methodology Research Group, School of Community Based Medicine, University of Manchester, Manchester, United Kingdom; Catholic University of Sacred Heart of Rome, ITALY

## Abstract

**Objectives:**

Although cannabis use is common in bipolar disorder and may contribute to worse clinical outcomes, little is understood about the relationship between this drug and bipolar disorder over the course of daily life. The aim of study was to examine the effect of cannabis on affect and bipolar symptoms in a group of individuals with bipolar disorder.

**Methods:**

Twenty-four participants with bipolar disorder type I or type II completed diaries for 6 days using Experience Sampling Methodology to investigate the temporal associations between cannabis, affect and bipolar disorder symptoms.

**Results:**

The results indicated that higher levels of positive affect increase the odds of using cannabis (OR:1.25 ,CI:1.06–1.47, P=0.008). However, neither negative affect, manic nor depressive symptoms predicted the use of cannabis. Cannabis use was associated with subsequent increases in positive affect (β=0.35, CI:0.20-0.51, P=0.000), manic symptoms (β=0.20,CI:0.05-0.34, P=0.009) and depressive symptoms (β= 0.17,CI:0.04-0.29, P=0.008).

**Conclusion:**

The findings indicate that cannabis use is associated with a number of subsequent psychological effects. However there was no evidence that individuals with BD were using cannabis to self-medicate minor fluctuations in negative affect or bipolar disorder symptoms over the course of daily life. The findings in relation to existing literature and clinical implications are discussed.

## Introduction

Cannabis is the street drug most frequently used by individuals with bipolar disorder (BD) [1, 2, 3]. Estimates of current use range from 8% to 22% and lifetime use from 30% to 64% [4]. Cannabis use in BD is associated with poorer outcomes, including increased symptom severity [5] and poorer treatment compliance [5, 6]. A recent study found that individuals who were diagnosed with BD and a co-occurring cannabis disorder had a younger age of BD onset and an increased number of manic, hypomanic and depressive episodes per year [7].

Specific reasons for the high levels of cannabis use in BD remain equivocal and are not yet fully understood. Whilst there have been a number of reviews reporting on the co-occurrence of substance use and BD [8] few studies have focused specifically on the relationship between cannabis use and BD [7].

Prospective cohort studies [9, 10, 11] have found evidence to suggest that cannabis use begins prior to bipolar onset, which might suggest a causal role in the development of BD. However there is also evidence to suggest that for some, cannabis use commences following the onset of manic symptoms [12]. Self-report literature including case histories [13, 14] and qualitative interviews [15] suggests that individuals with BD use cannabis as a form of self-medication to alleviate manic symptoms [13, 15] and to relieve depression [14]. These findings [13, 14, 15] are consistent with the proposal of Ashton et al [16] that the key consitutents of cannabis, ∆9-tetrahydrocannabinol (THC) and cannabidiol (CBD), can have both sedative and antidepressant effects. Therefore whilst manic, individuals may use cannabis for the sedative effects and when depressed for the anti-depressive effects.

As with BD, rates of cannabis use in individuals with psychosis are high [17, 18] and there is no single model available which fully explains this co morbidity [19]. A recent study [20] used the experience sampling method (ESM) to provide further insight into the complicated dynamics of cannabis use and its effect on individuals with psychosis, in the context of daily life. Henquet et al [20] found that cannabis use predicted an increase in positive affect in both individuals with psychosis and a non-clinical control group. Cannabis use also predicted a decrease in negative affect and an increase in the number of hallucinatory experiences in the psychosis group alone. They found no evidence to support the self-medication hypothesis as neither psychotic experiences or mood was found to predict cannabis use. The current study was similarly designed using ESM to allow a close investigation into BD and cannabis use over the course of daily life and to aid further understanding of this seemingly complex relationship.

ESM is a structured diary method where individuals are asked to report their thoughts, feelings and symptoms over the course of daily life. ESM was pioneered in mental health research by researchers at the University of Maastricht [21, 22]. The methodology offers a number of advantages in comparison to traditional assessments of mental health experiences [23, 24], which rely on using retrospective data, and may be open to recall bias. With ESM, the short space between an event occurring and reporting of the details reduces the possibility of memory bias [25]. ESM examines phenomena in the real world as they occur and therefore has a high level of ecological validity. It provides a rich and descriptive data set, detailing a participant’s daily experience and has the capacity to assess the temporal relationship between numerous variables [24].

ESM has previously been used to investigate the perception of daily ‘hassles’ and ‘uplifts’ in individuals with BD [26]. The study found that individuals who had more previous depressive episodes and current depressive symptoms experienced negative events as more stressful [30]. Furthermore, Knowles et al [27] used a diary method, where individuals with remitted BD or remitted unipolar depression plus a non-clinical sample reported on self-esteem and positive and negative affect twice a day, over the course of a week. The study reported instability of self-esteem and affect in the remitted BD individuals compared to both other groups.

To the authors knowledge there are no published studies that have used ESM to examine the relationship between BD and cannabis use. Therefore the current study aimed to provide an investigation into the associations between cannabis use, positive affect, negative affect and BD symptoms (mania and depression) in individuals with BD in daily life over a six day period, and to test two key predictions suggested in the literature outlined above:

1] frequency of cannabis use will increase as a function of affect and BD symptom change (i.e. self medication effects)
2] cannabis use will be associated with subsequent changes in affect and BD symptoms

## Materials and Methods

### Ethics statement

Full ethical approval for the study was obtained from Liverpool National Research Ethics Service (NRES) Committee and the University of Manchester research ethics committee. Written, informed consent was obtained from all participants in the study.

### Participants

Twenty-nine participants were recruited for the study from a number of sources. These included four mental health trusts in the North-West of England, self-help organisations (Bipolar UK and Mood Swings Network) and self-referral from the online University of Manchester research volunteering website (http://www.studentnet.manchester.ac.uk/volunteer/).

All individuals met criteria for BD-I or BD-II, as determined by the Structured Clinical Interview for Axis I Disorders (SCID) based on the DSM-IV diagnostic criteria [28]. Current symptomatology was assessed using the Hamilton Rating Scale for Depression [29] and the Bech Rafaelson Mania Rating Scale [30]. Substance use disorders were assessed using the substance use module of the SCID [28]. To be included, participants were required to report using cannabis on at least two occasions per week (in at least half the weeks in the 3 months prior to assessment). Exclusion criteria for the study included meeting criteria for a current episode of mania or depression (if currently met criteria they were kept on a waiting list until out of episode, except for those who remained unwell throughout the recruitment period), aged below 18, evidence of an organic brain disease or moderate/severe learning disability.

### Experience sampling method and procedure

At the beginning of this study, participants were given a paper diary and a digital wristwatch. In accordance with previous research [31, 32], the ESM period lasted for six consecutive days and the watch emitted a signal on ten occasions throughout the day at unpredictable times, between the hours of 8am and 10pm.

Each time participants heard the beep they were required to fill out a page of the diary. The diary consisted of questions on thoughts, affect, BD symptomatology, contextual information regarding their current situation and substance use. Participants were required to fill out the diary within 15 minutes of hearing the beep and to record the time of completion. Any entries completed outside this time frame were excluded from analyses. Previous research has demonstrated that entries completed after the 15 minutes are less reliable and valid [22]. A minimum of 20 valid diary reports were required by each participant, to ensure the data was representative [24].

During the initial visit informed consent was gained from the participant and the SCID [28], Hamilton Rating Scale for Depression [29] and the Beck Rafaelson Mania Rating Scale [30] were completed. Where all inclusion criteria were met, a second visit was arranged, one day prior to the ESM period. During the second visit, the participant was introduced to the watch and paper diary and briefed about the study. The general procedure described above was explained in detail. On the seventh day a final meeting was arranged to collect the watches and diaries and debrief the participant.

## Measures

### The ESM diary—affect items

Current affect was assessed using ten items, rated on a 7-point Likert scale (where 1 = ‘not at all’ and 7 =’ very much so’). In previous ESM studies [20,31,33], with individuals with psychosis and healthy controls, a positive and negative affect scale was identified using a factor analysis on the raw-within participant scores for the affect items (N = 10). A principal components analysis for this study was conducted and like the similar studies [20,31,33] revealed two separate scales. The items ‘cheerful’, ‘excited’, ‘relaxed’, ‘satisfied’, ‘happy’ formed the positive affect scale (∝ = 0.85) and the items ‘lonely’, ‘anxious’, ‘irritated’, ‘sad’, ‘guilty’, formed the negative affect scale (∝ = 0.82). This analysis yielded similar results to other ESM studies. Henquet et al [20] reported a positive affect scale (∝ = 0.89) and a negative affect scale (∝ = 0.80). The mean scores for each scale were used in the analyses.

### The ESM diary—BD Symptoms

Current BD symptomatology (mania and depression) was assessed using 7 items rated on a 7-point Likert scale. The items were formulated by the authors (SJ, CB, ET), all of whom had significant experience of working with people with BD. They were chosen in accordance with guidelines for selection of ESM items [24] and to assess momentary experiences of BD symptoms that might reasonably occur and fluctuate during the flow of daily life. A service-user group of 4 people with a BD diagnosis took part in a consultation exercise and checked the appropriateness of the potential questions. Members reported that the language reflected how they would describe their own behaviour and experiences. A principal components analysis revealed two distinct subscales with high internal consistency. The mania scale (∝ = 0.76) consisting of the items: I am ‘full of energy’, ‘high’, ‘ full of good ideas’ and the depression scale (∝ = 0.83), consisting of items: I feel ‘slowed down’, ‘low’, ‘bad about myself’, ‘fearful’. The mean scores for each scale were used in the analyses.

### The ESM diary—substance use

Cannabis use, referred to as a ‘cannabis moment’ was reported in the diary after each beep (the period between the current beep and previous beep). Information was derived from the question (since the last beep I’ve used cannabis’Yes’ or ‘No’?) Cannabis use **previous** was defined as cannabis use during the period between previous beep and the beep before that. The type of cannabis used was also recorded (skunk, resin or grass).

Alcohol and drug use other than cannabis were reported in the diary after each beep, termed ‘alcohol moment’ and ‘other drug moment’ respectively.

### Statistical analysis strategy

STATA 11 [34] was used for the analyses. ESM data has a hierarchical structure with the repeated participant observations (level one), nested within days (level two), nested within participants (level three). Responses for one individual or for one day are more likely to be similar than those for a different individual or for a different day. Multilevel random regression analysis was used as it takes the whole data set into account and can estimate the amount of variation that is associated with the three different levels. The multilevel regression XTMELOGIT routine was used for the dichotomous variables and the XTMIXED routine for the continuous variables. Therefore the odds ratios (dichotomous variables) and the betas (continuous variables) are the associations between the independent and dependent variables in the multilevel model.

### Preliminary analyses

Multi-level regression analyses were conducted to identify whether age, gender, alcohol use at the same beep, other drug use at the same beep, type of cannabis used and total cannabis use for the ESM period were associated with changes in affect, BD symptoms and cannabis use. The results were used to identify which variables would be adjusted for in the main analyses.

### Self medication effects

To investigate whether affect or BD symptoms predicted cannabis use; multilevel analyses were conducted using the XTMELOGIT routine. Positive affect **previous**, negative affect **previous**, mania **previous** and depression **previous** were entered as the independent variables and cannabis use as the dependent variable. Overall cannabis use during the ESM week was adjusted for in these analyses.

### Cannabis effects on affect and BD symptoms

The main effects of cannabis use on affect / symptoms were investigated with cannabis use as the independent variable and positive affect, negative affect, mania and depression as the dependent variables. Alcohol use at the same beep and overall cannabis use during the ESM week were adjusted for in these analyses.

### Temporal analyses of cannabis use

Post hoc analyses were conducted to further investigate the duration of cannabis effects on affect and symptoms. To investigate these, cannabis use at the current beep and cannabis use **previous** were entered simultaneously into the model, predicting positive affect, negative affect, mania and depression.

## Results

### Participants

Twenty-nine participants initially participated in the study. However three participants were subsequently excluded as they had fewer than 20 valid reports and a further two dropped out due to personal circumstances. The final study sample consisted of 24 participants. See table 1 for socio-demographics of the participant sample.

**Table 1:** Socio-demographics of the participant sample.

**Gender** (F:M)	8:16
**Age** Mean (SD)	37.1 (12.6)
**Diagnosis** (BDI: BD II)	22.2
**Current HAM** mean (SD)	7.7 (7.2)
**Current MRS** mean (SD)	2.9 (3.3)
**Ethnicity**	
White British	21 (88%)
Other White Background	1 (4%)
Black Caribbean	1 (4%)
White and Asian	1 (4%)
**Living status**	
Living alone	12 (50%)
Living with friends	5 (21%)
Living with partner and/or children	6 (25%)
Living with close relative	1 (4%)
**Occupation**	
Sick/ Disability	14 (58%)
Student	4 (17%)
Employed/ self employed	3 (13%)
Employed voluntary	2 (8%)
Unemployed	1 (4%)
**Medication**	
Prescribed mood stabilizer	20 (83%)
No mood stabilizer	4 (17%)
**Co-morbidity (current)**	
Anxiety disorders *	6 (25%)
Personality disorders **	5 (21%)

* Anxiety disorders included panic disorder (with and without agoraphobia), generalised anxiety disorder, obsessive compulsive disorder, post-traumatic stress disorder, social phobia, specific phobia.

** Personality disorders included Borderline personality disorder and anti-social personality disorder

### Participant exclusion /drop outs

Five participants were excluded / dropped out of the study. There were no significant differences between individuals who completed the study and those who did not in relation to gender, ethnicity, bipolar diagnosis, marital status, occupational status or living arrangements. However significant differences were revealed between the two groups in terms of age. Individuals who were excluded/ dropped out had a higher mean age and scores on the HAM and the MAS were elevated for the group that was excluded/ dropped out (see table 2 for actual values).

**Table 2:** Socio-demographics of the dropped out / excluded sample.

**Gender (F:M)**	1:4
**Age** Mean (SD)	44.6 (12.1)
**Diagnosis** (BDI: BD II)	5:0
**Current HAM** mean (SD)	8.25 (8.5)
**Current MRS** mean (SD)	4.25 (3.1)
**Ethnicity**	
White British	5 (100%)
Other White Background	0
Black Caribbean	0
White and Asian	0
**Living Status**	
Living alone	3 (60%)
Living with friends	0
Living with partner and / or children	2 (40%)
Living with close relative	0
**Occupation**	
Sick / Disability	5 (100%)
Student	0
Employed/ self-employed	0
Employed voluntary	0
Unemployed	0

### Substance use

Three participants met current criteria for cannabis abuse disorder and 12 met criteria for current cannabis dependence disorder. Over the course of the six-day ESM period, the mean number of cannabis moments for the sample was 15.0 (SD: 8.6, range: 2–30). During this period all participants reported only using one type of cannabis, with the majority using ‘skunk’ (54%). The mean number of alcohol moments for the sample was 3.1 (SD: 5.5, range 0–21) and for ‘other’ drug use 0.8 (S.D: 2.5, 0–12). See table 3 for other drug and alcohol use.

**Table 3:** Substance abuse in the participant sample.

Cannabis	
**Current abuse**	3 (13.0%)
Current dependence	12 (50%)
**Type of cannabis used**	
Skunk	13 (54%)
Resin	8 (33%)
Grass	3 (13%)
**Cannabis moments over ESM period**	
Mean (S.D)	15.0 (8.6)
Range	2–30
**Alcohol**	
Current abuse	2 (8%)
Current dependence	1 (4%)
**Alcohol moments over ESM period**	
Mean (S.D)	3.1 (5.5)
Range	0–21
**Other drug use**	
Current abuse	2 (8%)
Current dependence	1 (4%)
**Other drug moments over ESM period**	
Mean (S.D)	0.8 (2.5)
Range	0–12

### Preliminary analyses

Age, gender, other drug use (at the same beep) and type of cannabis used were not associated with any of the outcome variables, therefore were not adjusted for in the main analyses. Total cannabis use for the ESM period (number of cannabis moments) was positively associated with cannabis use at the current beep (β = 0.12, 95% CI: 0.10–0.15, P = 0.000), therefore this was adjusted for in all the main analyses. Alcohol use (at the same beep) was positively associated with subsequent increases in positive affect (β = 0.48, 95% CI: 0.21–0.74, P = 0.000) and manic symptoms (β = 0.40, 95% CI: 0.15–0.65, P = 0.002), therefore it was adjusted for in the analyses which investigated the effects of cannabis.

### Self—medication effects

There was a significant positive relationship between positive affect **previous** and cannabis use at the current beep (OR: 1.25, 95% CI: 1.06–1.47, P = 0.008). The odds of cannabis use at the current beep were increased for those with higher scores of positive affect at the previous beep. Negative affect **previous** did not significantly predict cannabis use at the following beep (OR: 0.88, 95% CI: 0.74–1.05, P = 0.147). Similarly no association was found between manic symptoms **previous** (OR: 1.08, 95% CI: 0.93–1.26, P = 0.291) or depressive symptoms **previous** (OR: 0.92, 95% CI: 0.78–1.08, P = 0.303) and cannabis use. See table 4.

**Table 4:** Effect of mood/ BD symptomatology on cannabis use (Self-medication effects).

Positive affect	OR: 1.25, 95% CI: 1.06–1.47, P = 0.008
Negative affect	OR: 0.88, 95% CI: 0.74–1.05, P = 0.147
Mania scale	OR: 1.08, 95% CI: 0.93–1.26, P = 0.291
Depression scale	OR: 0.92, 95% CI: 0.78–1.08, P = 0.303

### Cannabis effects on affect and BD symptoms

Cannabis use was associated with subsequent increases in positive affect (β = 0.35, 95% CI: 0.20–0.51, P = 0.000). Cannabis use was also associated with subsequent increases in manic symptoms (β = 0.20, 95% CI: 0.05–0.34, P = 0.009) and depressive symptoms (β = 0.17, 95% CI: 0.04–0.29, P = 0.008). Overall, cannabis use had no effect on negative affect (β = -0.01, 95% CI: -0.13–0.10, P = 0.806). (See table 5).

**Table 5:** Effect of cannabis use on mood/ BD symptoms.

Positive affect	β = 0.35, 95% CI: 0.20–0.51, P = 0.000
Negative affect	β = -0.01, 95% CI: -0.13–0.10, P = 0.806
Mania Scale	β = 0.20, 95% CI: 0.05–0.34, P = 0.009
Depression Scale	β = 0.17, 95% CI: 0.04–0.29, P = 0.008

### Temporal dynamics of cannabis effects

Follow up post–hoc analyses were conducted to investigate the duration of cannabis effects on affect and BD symptoms. This was achieved by entering cannabis use and cannabis use **previous** simultaneously into the model. The results suggested that increases in positive affect were observed in the short term (β = 0.29, 95% CI: 0.10–0.48, P = 0.003 for cannabis use) but not the longer term (over one beep but not two) (β = 0.01, 95% CI: -0.18–0.20, P = 0.943 for cannabis use **previous**). Similarly increases in depressive symptoms were observed in the short term (β = 0.18, 95% CI: 0.03–0.33, P = 0.019 for cannabis use) but not the long term (β = 0.11, 95% CI: -0.04–0.27, P = 0.138 for cannabis use **previous**). For mania, when both cannabis use and cannabis use **previous** were entered simultaneously in the same model, increases in manic symptoms were not observed in the short term (β = 0.07, 95% CI: -0.10–0.24, P = 0.393) or long term (β = -0.08, 95% CI: -0.25–0.09, P = 0.359). See table 6.

**Table 6:** Temporal dynamics of cannabis effects.

**Positive affect**	
Cannabis use	β = 0.29, 95% CI: 0.10–0.48, P = 0.003
Cannabis use **previous**	β = 0.01, 95% CI: -0.18–0.20, P = 0.943
**Negative affect**	
Cannabis use	β = -0.04, 95% CI: -0.18–0.10, P = 0.579
Cannabis use **previous**	β = -0.01, 95% CI: -0.15–0.13, P = 0.925
**Mania Scale**	
Cannabis use	β = 0.07, 95% CI: -0.10–0.24, P = 0.393
Cannabis use **previous**	β = -0.08, 95% CI: -0.25–0.09, P = 0.359
**Depression Scale**	
Cannabis use	β = 0.18, 95% CI: 0.03–0.33, P = 0.019
Cannabis use **previous**	β = 0.11, 95% CI: -0.04–0.27, P = 0.138

## Discussion

Higher levels of positive affect increase the odds of using cannabis, however neither negative affect, manic symptoms nor depressive symptoms predicted the use of cannabis at the subsequent beep. The data from the current study indicates that individuals with BD are not using cannabis to self-medicate minor fluctuations in negative affect and bipolar symptoms (previous beep to current beep). It remains to be seen how this data relates to the broader self-medication hypothesis in BD. In line with the second prediction, the findings from the study indicate that the use of cannabis in daily life was associated with subsequent increases in positive affect, manic symptoms and depressive symptoms. In addition, the data suggests that increases in positive affect and depressive symptoms were only experienced in the short-term, as cannabis use at the previous beep did not predict a significant increase in affect or symptoms at subsequent time points.

### Cannabis effects

The findings that cannabis use was associated with an increase in positive affect, manic and depressive symptoms is consistent with current literature that suggests cannabis can produce a range of psychological effects [35, 36, 37]. It has been suggested that the psychological and physiological effects of cannabis are primarily due to its main chemical compounds, THC and CBD. The effects of cannabis have previously been found to be bidirectional [35, 38], causing effects such as euphoria and dysphoria; this may partially explain why cannabis use was associated with both manic and depressive symptoms in the current study. The bidirectional effects of cannabis have been found to depend on a range of factors such as dose, route of administration and personality differences [35, 38].

The effect of cannabis use on individuals with psychosis has received rather more investigation than the effects of the drug on those with BD. Research suggests that compared to ‘healthy’ control participants, individuals with at high risk for psychosis may be more sensitive to THC [39, 40]. Barkus and Lewis [41] found that individuals scoring higher on Schizotypal traits were more likely to experience both psychosis-like experiences and more pleasurable experiences after smoking cannabis. Individual differences in sensitivity to the effects of THC may explain the range of experiences, as noted by Henquet et al [20]. In a similar way, individuals with BD may also differ in their sensitivity to the effects of THC, which may explain why there was a range of effects in the current study.

### Effects of affect / BD symptoms on cannabis use (Self-medication effects)

The results indicated that higher levels of positive affect increase the odds of using cannabis, and it appears that individuals were using cannabis when they were feeling good. Alternatively, higher positive affect prior to cannabis use may have been experienced due to the expected enjoyment of the effects of substance use.

Data from the current study does not support the idea that cannabis is used by individuals with BD for the self-medication of minor fluctuations in negative affect and BD symptoms in the context of daily life. An increase in negative affect and BD symptoms did not predict cannabis use at the following beep. This finding is consistent with Henquet et al [20] who similarly did not find evidence to support the self-medication hypothesis for psychosis over the course of daily life, as changes in hallucinations, delusions and negative affect did not predict cannabis use.

The interpretation of the findings of this study is limited to the associations between the current beep and the previous beep. It is possibly the case that self-medication effects appear further down the chain of events, following a longer period of negative affect / symptom changes. Alternatively, failure to find self-medicating effects from cannabis may have been due to the nature of the participant sample. BD is characterised by shifts in affect regulation and therefore over time individuals may have become accustomed to subtle changes in mood. Therefore, within the context of daily life, cannabis may not be used as a way to cope with these slight fluctuations. Participants in the study were currently well and out of episode and therefore it may be that cannabis is used to self-medicate more pronounced symptoms or the onset of manic / depressive episodes. This would be consistent with the self- report literature where individuals have found cannabis useful in the management of their BD [13, 14, 15].

## Limitations

Several limitations need to be taken into account in interpreting the results of the study. First, details of cannabis use were based on self-report. Cannabis use remains illegal in the United Kingdom, and this may have led to underestimations in reported use. Hair sample analysis may have offered a way to confirm usage [42], however this was beyond the resources of the current study. Additionally, whilst type of cannabis was reported and adjusted for in analyses, the individual potency of the drugs consumed was not controlled for. There are in excess of 100 different strengths of cannabis and research has revealed that on average, cannabis resin and herbal (grass) contains around 2–4% THC, however Sinsemillia (Skunk) contains around 12–18% THC [43, 44]. Data for cannabis use at each bleep was dichotomized into ‘yes’ or ‘no’; future studies might attempt to collect and report information regarding the amount of cannabis ingested and route of consumption at each beep.

The items used on the scales for mania and depression were formulated specifically for use in the ESM diary in this study. They were chosen in accordance with guidelines for the selection of ESM questions [24] which highlights the need for items to be ‘momentary experiences which occur in the flow of daily life’. Whilst we have limited evidence for their validity, items were reviewed by a service user panel with BD who felt they accurately described their experience when manic and depressed. A correlation matrix was computed to investigate the relationship between the positive affect, negative affect, mania and depressive scales. There was a strong correlation between the negative affect and depressive scales (r = 0.82). This indicates that there may have been a high degree of overlap between the scales. The correlation between the positive affect scale and mania scale was (r = 0.58). However during the main analyses the scales produced different results (e.g. cannabis use was significantly associated with depressive symptoms but not with negative affect and higher levels of positive affect increase the odds of using cannabis, however higher levels of manic symptoms did not). This provided some predictive validity to support the use of separate scales: and it suggests that they were measuring different emotional states. Items from the positive and negative affect scales were considered to reflect everyday mood fluctuations as expected within the ‘normal’ range. The items formulated for the mania and depression scales were deemed to reflect symptoms specific to BD that would fluctuate over the course of daily life.

Cannabis is known to have an impact on cognition [45, 46] and as suggested by Henquet et al [20] this may therefore have impacted on the ability to report information accurately in the diaries. However one of the main advantages of using ESM is the short space of time between an event occurring and the recall, which reduces memory bias [25]. Additionally, a recent study [47] found cannabis use was associated with better neuro-cognitive functioning in participants with BD, particularly executive functioning.

Cannabis is rapidly metabolized in the body and the pharmacological effects can often begin within minutes after smoking [48]. Blood plasma levels of THC peak approximately 20 minutes after ingestion [49]. However traces of THC can exist in the body for several days following use. There may have been a background level of cannabis in the system for some of the regular users taking part in the study and this must be taken into account when interpreting the results. However, the consequences produced from ‘re-dosing’ during the cannabis moments are still valid due to the almost immediate effects of cannabis use.

ESM can be a demanding methodology and requires sustained attention and motivation to fill out diary entries. This may deter some individuals, and thereby result in a selection bias. In addition, like other ESM studies [50, 51] the participant sample size was relatively small and therefore may not generalize to all individuals with co-occurring cannabis use and BD. However a large number of data points were generated as ESM data has a hierarchical structure with the repeated participant observations (level one), nested within days (level two), nested within participants (level three).

Additionally, the majority of the sample was from a white British background and had a diagnosis of BD-I. It is therefore questionable how much the findings of this study may generalise to people from different ethnic minorities or other BD groups.

Finally, Henquet et al [20] used a study sample with individuals with a clinical diagnosis of a psychotic disorder and healthy controls. Both groups were frequent cannabis users (current use of at least 3 times per week). The results from the study were different for both groups, with cannabis use associated with a decrease in negative affect and an increase in hallucinatory experiences in the clinical group alone. The inclusion of a control group (individuals without a mental health diagnosis who regularly used cannabis) in this study may have provided insight into whether the findings of the study relate exclusively to those with a diagnosis of BD, compared to a non-clinical sample.

## Clinical Implications

Overall results from the present study indicate that cannabis use can cause a range of psychological effects for individuals with BD, including an exacerbation of both manic and depressive symptoms. Co-occurring BD and substance abuse is highly prevalent [6, 52] and it is associated with worsened outcomes [5, 6, 7]. However intervention research for BD and substance abuse is in its infancy [53, 54, 55, 56] and demonstrates a limited evidence base [53]. The results from this study may help to inform future interventions.

Clients often find it difficult to reduce their substance intake and the literature suggests that some individuals perceive cannabis as a useful coping strategy in the management of their BD symptoms. However results from this study may help to counter these positive expectations of their substance use. The findings suggest that cannabis is not being used to self medicate changes in symptoms, within the context of daily life, and in fact it may be further complicating affective states. Services and clinicians need to be aware of the potential impact of using cannabis and able to inform clients of the risks. Alongside this it may be helpful for clinicians to offer alternative strategies to help clients cope with changes in BD symptoms, which may in turn increase an individual’s confidence to reduce their substance intake.

Similar to Henquet et al’s findings [20], the majority of participants in this study reported that they found the ESM diary a useful and reflective tool to monitor their mood and cannabis use. Anecdotally, a number of participants reported that tracking patterns of mood and cannabis use led them to question their substance use and in some cases reduce intake. ESM could provide an invaluable therapeutic tool, particularly with clients who are ambivalent about changing their drug use habits, providing insight into unhelpful patterns of behaviour, which may contribute towards the maintenance of their difficulties.

## Conclusion

The findings from the study indicate that cannabis use is associated with a subsequent change in positive affect, depressive symptoms and manic symptoms over the course of daily life. No evidence for the use of cannabis to self-medicate minor fluctuations in negative affect or BD symptoms was revealed. Participants in the study were currently well and out of episode. Future research should explore whether the self-medication hypothesis is more relevant to individuals that are in the acute stages of depression or mania. This would be consistent with the broader self-medication hypothesis in BD where individuals have reported finding cannabis useful in the management of their symptoms [13, 14, 15].

## Supporting Information

S1 Dataset (SAV)
