# Correction: The Relationship between Bipolar Disorder and Cannabis Use in Daily Life: An Experience Sampling Study

**DOI:** 10.1371/journal.pone.0123953

**Published:** 2015-03-30

**Authors:** The PLOS ONE Staff

There are errors in the Funding section. The correct funding information is as follows: The study was funded primarily through the University of Manchester, UK Doctorate of Clinical Psychology training course. NB contributed towards this as part of her MPhil studies whilst employed as a research assistant by the PARADES programme grant (RP-PG-0407-10389) led by Professor Steven Jones and funded by National Institute for Health Research (NIHR), UK The funders had no role in study design, data collection and analysis, decision to publish, or preparation of the manuscript.
